# Performance of the colorectal cancer screening marker Sept9 is influenced by age, diabetes and arthritis: a nested case–control study

**DOI:** 10.1186/s12885-015-1832-6

**Published:** 2015-10-29

**Authors:** Mai-Britt W. Ørntoft, Hans J. Nielsen, Torben F. Ørntoft, Claus L. Andersen, On behalf of the Danish Study Group on Early Detection of Colorectal Cancer

**Affiliations:** Department of Molecular Medicine, MOMA, Aarhus University Hospital, Skejby, DK-8200 Aarhus N Denmark; Department of Surgical Gastroenterology 360, Hvidovre Hospital, University of Copenhagen, DK-2650 Hvidovre, Denmark

**Keywords:** 3–10 words: Sept9, Colorectal cancer, Screening, DNA methylation marker, Comorbidities, Epigenetics

## Abstract

**Background:**

Annually, colorectal cancer (CRC) is diagnosed in >1.4 million subjects worldwide and incidence is increasing. Much effort has therefore been focused on screening, which has proven to reduce cancer-related mortality. The Sept9 DNA-methylation assay is among the most well studied blood-based screening markers. However, earlier reported performances may be misleading: the Sept9 test was recently examined in two screening based cohorts and yielded performances lower than expected. We hypothesize that comorbidities and/or demographic characteristics affect the results of the Sept9 test.

**Methods:**

Using a retrospective nested case–control study design, we studied plasma from 150 cancer and 150 controls selected from a well-characterized cohort of 4698 subjects referred for diagnostic colonoscopy due to CRC-related symptoms. The cases and controls were matched on age and gender, and moreover cases were stratified on tumor-site and tumor-stage. The selected cohort included a wide range of comorbidities. Plasma Sept9 levels were assessed using a commercially available PCR based assay (Epi-proColon).

**Results:**

Clinical sensitivity for CRC stages I-IV was 37 %, 91 %, 77 %, and 89 %, and the overall sensitivity 73 % (95 % CI, 64–80 %) and specificity 82 % (95 % CI, 75–88 %), respectively. Age >65 was associated with both increased false positive and false negative results (*p* < 0.05). Arthritis was associated with a higher false negative rate (*p* = 0.005) whereas Arteriosclerosis was associated with a higher false positive rate (*p* = 0.007). Diabetes was associated with Sept9 positivity with an OR of 5.2 (95 % CI 1.4–19.1). When the performance of Sept9 was adjusted for these parameters in a final multivariate regression model, the OR for a positive Sept9 test to be associated with CRC increased from 8.25 (95 % CI 4.83–14.09) to 29.46 (95 % CI 12.58–69.02).

**Conclusions:**

The results indicate that the performance of the Sept9 assay is negatively affected by several factors commonly associated with CRC screening populations: early-stage disease, age > 65 years, diabetes, arthritis, and arteriosclerosis. This should be taken into account if the Sept9 assay is used as a single marker for CRC screening, but may also have a wider impact, as it is likely that such factors may affect other blood based DNA markers as well.

**Electronic supplementary material:**

The online version of this article (doi:10.1186/s12885-015-1832-6) contains supplementary material, which is available to authorized users.

## Background

At present, primary colorectal cancer (CRC) is diagnosed in >1.4 million subjects annually and incidence is increasing [1]. Although improved surgical approaches for especially rectal cancer has improved the overall survival rates it is mainly early stage cancers that are curable by surgical intervention. The most reliable self-reported symptom of CRC cancer is dark rectal bleeding with a positive predictive value of 20.6 % and an odds ratio (OR) of 7.4 for CRC [2], but symptoms may be evasive. Therefore, much effort has been focused on screening and hence earlier detection of CRC, which has repeatedly proven to reduce the cancer-related mortality [3, 4].

In recent years several screening markers have emerged to help diagnosing early stage CRC or even premalignant lesions. They separate in two different categories: stool markers, such as FOBT/FIT and blood-based markers as DNA/RNA and proteins [5]. It is expected that blood-based screening assays may improve the clinical sensitivity compared with stool tests, because of an expected higher compliance among screening subjects [6, 7]; however the cost-effectiveness has not yet been proven [8]. The Sept9 DNA-methylation test is among the most well studied blood tests; often in case–control study designs [9–11]. Recently, the Sept9 test was examined in two medium/large sized asymptomatic screening based cohorts and yielded overall sensitivities (range 48–68 %) and specificities (range 78–91 %) lower than expected based on the earlier case–control studies [12, 13]. This may indicate that the performance of the Sept9 test is affected by covariates, such as comorbidities and/or demographic characteristics of the subjects in screening populations. However, currently we have only limited knowledge of which comorbidities or characteristic could be involved as this has not been part of earlier study objectives.

To address this question we have tested the Sept9 assay on a nested case–control cohort selected from a large well-characterized cohort of subjects referred to colonoscopy due to CRC symptoms; the subjects also had a wide range of co-morbidities.

## Methods

### Human plasma samples

The retrospective nested case–control cohort in the present study was selected from the prospective Danish Endoscopy II study, a multicenter trial, which from April 2010 to November 2012 recruited 4698 subjects referred to colonoscopy due to CRC related symptoms. All patients gave informed consent to participate and the study was approved by the The Danish National Ethics Committee (H-3–2009–110) and the Danish Data Protection Agency (2007–58–0015). Exclusion criteria were previous colonoscopy, previous CRC or adenoma, diagnosis with HNPCC (Hereditary Nonpolyposis Colorectal Cancer or Lynch Syndrome) or FAP (Familial adenomatous polyposis), previous or present extracolonic malignant disease, or age under 18. Just prior to colonoscopy all subjects had a blood sample collected. All clinical information was collected, including surgical/oncological intervention, as well as cancer TNM stage, and adenoma histology. Intervention followed the Danish Colorectal Cancer Group (DCCG) guidelines. Chronic diseases were divided into large disease groups: Hypertension, Diabetes (type I and II), manifest Arteriosclerosis (pooling former AMI, stroke due to thrombosis, chronic ischemic diseases in peripheral arteries and chronic ischemic heart diseases), respiratory diseases and Arthritis (active arthritis in more than one joint).

The nested case–control cohort consisted of 300 participants: 150 cases and 150 controls with no evidence of CRC disease (NED). Cases consisted of 21 high risk adenomas (size ≥1 cm and/or villous histology >25 % and/or sessile-serrated polyps and/or high-grade neoplasia and/or ≥ 3 adenomas), 35 stage I CRC, 35 stage II CRC, 30 stage III CRC and 29 stage IV CRC based on UICC criteria. To minimize confounding, cases and controls were matched by age and gender. Cases were selected with gender evenly distributed according to disease stage and with 1/3 of tumors localized in the rectum, 1/3 in the proximal colon (coecum, ascending colon and right flexure), and 1/3 in the distal colon. Chronic disease and comorbidities were not included as exclusion criteria. For all participants, clinical follow up for further 3 years were obtained; as all Danish patients are registered with a personal computerized ID-number, and all hospital treatment is recorded in a national database, there were no participants lost to follow-up. A survey of the cohort identified the expected associations between co-morbidities and life style factors, indicating the cohort is representative of future CRC screening cohorts (Additional file 1: Table S1).

For all 300 participants, plasma was isolated from ethylenediaminetetraacetic acid (EDTA) stabilized whole blood by double centrifugation at 10 min for 3000 g at room temperature. Plasma was then stored at −80 °C under 24/7 electronic surveillance until isolation of circulating cell-free DNA (cfDNA).

### Sept9 test

cfDNA isolation and analysis for presence of methylated Sept9 DNA was done using the Epi-ProColon kit as described by Potter et al. [13]: The Epi proColon test comprises the Epi proColon Plasma Quick kit, the Sensitive PCR kit, and Control kit. All analyses were done blinded to subject outcome and were performed by Epigenomics GmbH, Berlin. Samples were processed in batches with a random distribution of cases and controls to avoid analytical bias, and negative and positive processing controls ensured validity of the test result. A minimum of 2 ml of plasma was provided for cfDNA isolation; one participant did not meet this requirement and was excluded. cfDNA was isolated from 3.5 ml plasma for 139 participants, and from between 2.0–3.5 mL plasma from 160 participants; a total of 299 subjects (149 cases and 150 controls).

To isolate cfDNA we used the Plasma Quick kit, where plasma was mixed with 3.5 ml of lysis buffer and incubated for 10 min, after which magnetic beads and absolute ethanol were added; the sample was incubated on a rotator for 45 min. Impurities were removed from the magnetic beads in a wash step. The purified DNA was then released from the beads in elution buffer and treated at 80 °C with a solution containing ammonium bisulfite for deamination of cytosine. The converted cfDNA was captured by use of magnetic beads, passed through a series of wash steps, and eluted in 60 μL buffer. Samples were then analyzed for presence of methylated Sept9 DNA with the Sensitive PCR kit on a 7500 Fast Dx Real Time PCR device (Life Technologies). The assay was designed as a duplex real-time PCR for the methylated Sept9 γ promoter and ACTB (actin, beta) as an internal reference to assess the integrity of each sample. PCR was performed in triplicate with 15 μL template DNA per well and run for 45 cycles. We recorded PCR results from the 7500 Fast Dx software for ACTB and methylated Sept9 for each of the triplicate reactions. The validity of each sample batch was determined on the basis of methylated Sept9 and ACTB cycle threshold (Ct) values for the positive and negative controls. Samples were only deemed valid if the ACTB control was positive in all three replicates (had amplification curves detected within 45 cycles). Sept9 test results for individual samples were scored positive, if a Ct value was detected within 45 cycles. Samples were scored negative when no methylated Sept9 Ct value was reported for any of the 3 valid PCR replicates.

### Statistical methods

We assessed the association in between all variables and also pair-wise between Sept9 and all other available variables using Fishers exact tests. All tests were two-sided, and *p* < 0.05 was considered statistically significant. Test for trend of increasing true positive results with increasing tumor stage was done using the Wilcoxon rank sum test. Univariate logistic regression models were used to assess the diagnostic power of all available variables for CRC. Bivariate models were used to assess the association between Sept9 and all other available variables, including if any of the available variables modified the effect of the Sept9 test. Finally a multivariate model was built to assess the adjusted diagnostic power of the Sept9 assay in the context of all the variables affecting or associated with it. All reported models passed the Hosmer-Lemeshow’s goodness of fit test. All assumptions for the different analyses were fulfilled. STATA V.12.1 (StataCorp LP, Texas, USA) were used for all statistical analyses.

## Results

### Clinical performance

An overview of the demographic and co-morbidity characteristics of the included subjects is provided in Table 1. In this study the overall sensitivity of the Sept9 test for detecting CRC was 73 % (95 % CI, 64–80 %) vs 59 % (95 % CI 50–67 %), using the 1/3 and 2/3 scoring algorithms, respectively. Clinical sensitivity for the individual CRC stages I-IV, using the 1/3 and 2/3 algorithms was as follows: 37 % vs 17 %; 91 % vs 74 %; 77 % vs 63 %; and 89 % vs 86 %. The sensitivity was significantly lower for stage I than for the higher stage tumors (Wilcoxon rank sum test, *p* < 0.001). As expected the two algorithms yielded significantly different sensitivity and specificity results (*p* < 0.001, Fisher’s exact test). For high risk adenomas the sensitivity was 14 % (95 % CI, 3–63 %) vs 0 % (95 % CI, 0–1.6 %), for the two algorithms. The positivity rates for adenomas were not different from the rates in the NED group, 18 % (95 % CI, 12–25 %) vs 5 % (95 % CI, 2–9 %), for the two algorithms (Table 2).

**Table 1:** Demographic distribution of the nested cohort from the Endoscopy II prospective sample collection

Variable		Total	CRC	Adenoma	NED
		299	128	21	150
Sex	Female	151	65 (51)	11 (52)	75 (50)
	Male	148	63 (49)	10 (48)	75 (50)
Age	≤65	183	74 (58)	11 (52)	98 (65)
	>65	116	54 (42)	10 (48)	52 (35)
Rectal bleeding	No	166	54 (42)	10 (48)	102 (68)
	Yes	133	74 (58)	11 (52)	48 (32)
Anemia	No	250	102 (80)	19 (90)	129 (86)
	Yes	49	26 (20)	2 (10)	21 (14)
Weightloss	No	211	81 (63)	17 (81)	113 (75)
	Yes	88	47 (37)	4 (19)	37 (25)
Altered Defaecation	No	126	58 (45)	12 (57)	56 (37)
	Yes	173	70 (55)	9 (43)	94 (63)
Abdominal Pain	No	172	72 (56)	17 (81)	83 (55)
	Yes	127	56 (44)	4 (19)	67 (45)
Abdominal mass	No	283	119 (93)	0	143 (95)
	Yes	16	9 (7)	0	7 (5)
Distention	No	241	103 (80)	21 (100)	117 (78)
	Yes	58	25 (20)	0 (0)	33 (22)
Hypertension	No	183	81 (63)	10 (48)	92 (61)
	Yes	116	47 (37)	11 (52)	58 (39)
Diabetes	No	280	117 (91)	16 (76)	147 (98)
	Yes	19	11 (9)	5 (24)	3 (2)
Arteriosclerosis	No	243	108 (84)	19 (90)	116 (77)
	Yes	56	20 (16)	2 (10)	34 (23)
Respiratory disease	No	267	117 (91)	21 (100)	129 (86)
	Yes	32	11 (9)	0 (0)	21 (14)
Arthritis	No	281	120 (94)	20 (95)	141 (94)
	Yes	18	8 (6)	1 (5)	9 (6)
Smoke#	No	123	47 (37)	12 (57)	64 (43)
	Yes		81 (63)	9 (43)	86 (57)
Alcohol##	Normal	245	103 (80)	15 (71)	127 (85)
	Abuse	54	25 (20)	6 (29)	23 (15)
BMI###	<18,5	10	4 (3)	1 (5)	5 (3)
	18,5 to 25	162	70 (55)	10 (48)	82 (55)
	25 to 30	94	39 (31)	7 (33)	48 (32)
	>30	33	15 (12)	3 (14)	15 (10)
Plasma volume¤	< 3.5 ml	160	70 (55)	6 (29)	84 (56)
	3.5 ml	139	58 (45)	15 (71)	66 (44)

Data are n (%)

# Former smokers and current smokers pooled

## Abuse: Women > 7 units per week, Men >14 units per week

### Underweight < 18,5, Normal 18,5–25, Overweight 25–30, Heavy overweight >30 ¤ median 3.0 ml, range (2–3.4 ml)

*NED* No Evidence of Disease

**Table 2:** Summary of Epi proColon test performance in a nested case-control cohort from the Endoscopy II population

1/3 algorithm				
Diagnosis	Subjects (*n*)	Positive (*n*)	Negative (*n*)	Fraction (95 % CI)
CRC	128	93	35	0.73 (0.64–0.80)
stage I	35	13	22	0.37 (0.21–0.55)
stage II	35	32	3	0.91 (0.76–0.98)
stage III	30	23	7	0.77 (0.58–0.90)
stage IV	28	25	3	0.89 (0.72–0.98)
Non-CRC	171	30	141	0.18 (0.12–0.24)
Adenoma	21	3	18	0.14 (0.03–0.63)
NED	150	27	123	0.18 (0.12–0.25)
2/3 algorithm				
CRC	128	75	53	0.59 (0.50–0.67)
stage I	35	6	29	0.17 (0.07–0.34)
stage II	35	26	9	0.74 (0.57–0.88)
stage III	30	19	11	0.63 (0.44–0.80)
stage IV	28	24	4	0.86 (0.67–0.96)
Non-CRC	171	7	164	0.04 (0.02–0.08)
Adenoma	21	0	21	0.00 (0.00–0.16)
NED	150	7	143	0.05 (0.02–0.09)

*NED* No Evidence of Disease

Fraction: Positive fraction detected

Difference in 1/3 vs 2/3 algorithm: Fischer’s Exact Test, *p*< 0.001

Increasing proportion of true positive results with increasing tumor stage: Wilcoxon rank sum test for trend, z<0.001

The overall test specificity was 82 % (95 % CI, 75–88 %) vs. 95 % (95 % CI, 91–98 %) for the two algorithms. To investigate if any subjects with NED falsely scored positive due to an occult cancer, all subsequent instances of cancer diagnoses three years after the initial colonoscopy were identified through hospital records. None of the subjects with NED that scored falsely positive were later diagnosed with cancer.

### Factors potentially affecting assay performance

**Fig. 1:**
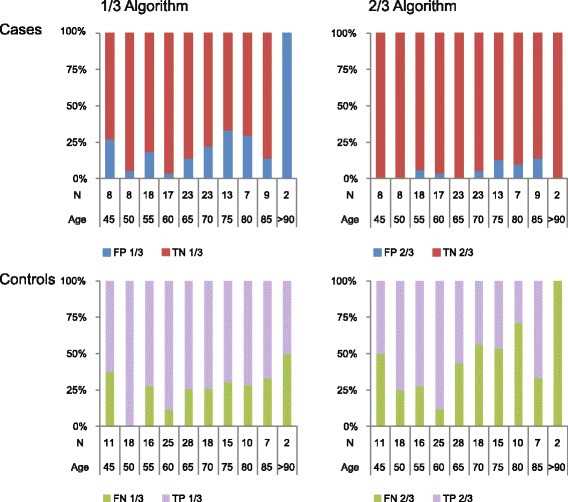
Age plotted against Sept9 outcome. FP: False positive, TN: True negative, FN: False negative, TP: True positive. N: Number of subjects in each category. Age: All ages younger or equal to the age interval mentioned. -1/3 and 2/3 refers to the PCR-algorithms used

To investigate if the outcome of the Sept9 test was affected by any of the available demographic or clinical variables (excluding symptoms) the significance of all associations with Sept9 was tested using Fisher’s exact test. Initially the continuous variable age was plotted against Sept9 outcome to look for trends of association, and to determine a cut-point for dichotomization of the age variable (Fig. 1). The plot revealed a tendency towards an increased false positive rate for NEDs with ages above >65 years, particularly for the high specificity 2/3 algorithm (Fig. 1, right panel). After dichotomizing age at 65 years and testing for association to Sept9, the group with age >65 was significantly associated with increased false positive rates for both the 1/3 and 2/3 algorithms (*p* = 0.015 and *p* = 0.05 respectively). The analysis also revealed that age >65 was associated with an increased false negative rate for the 2/3 algorithm (*p* = 0.007, Tables 3 and 4). However, this significance was probably driven by an unintended imbalance in tumor stage distribution in the two age groups, with the >65 age group having significantly fewer stage III and IV tumors (Additional file 2: Table S2).

**Table 3:** Sept9 positivity of individuals with CRC

		1/3 algorithm				2/3 algorithm			
Variable		*P*	*N*	% (95 % CI)	p*	*P*	*N*	% (95 % CI)	p*
		93	35	73	NR	75	53	59	NR
Sex	Female	42	23	65 (52–76)	0.05	34	31	52 (40–65)	0.16
	Male	51	12	81 (69–90)		41	22	65 ( 52–77)	
Age	≤65	57	17	77 (66–86)	0.23	51	23	69 (59–81)	0.007
	>65	36	18	67 (53–79)		24	30	44 (31–59)	
Hypertension	No	60	21	74 (63–83)	0.68	49	32	60 (49–71)	0.58
	Yes	33	14	70 (55–83)		26	21	55 (40–70)	
Diabetes	No	84	33	72 (63–80)	0.73	67	50	57 (48–66)	0.36
	Yes	9	2	82 (48–98)		8	3	73 (39–94)	
Arteriosclerosis	No	80	28	74 (65–82)	0.42	65	43	60 (50–69)	0.46
	Yes	13	7	65 (41–85)		10	10	50 (27–73)	
Respiratory disease	No	87	30	74 (65–82)	0.17	71	46	61 (51–70)	0.20
	Yes	6	5	55 (23–83)		4	7	36 (11–69)	
Arthritis	No	91	29	76 (61–83)	0.005	73	47	61 (52–70)	0.07
	Yes	2	6	25 (3–65)		2	6	25 (3–65)	
Smoke#	No	38	9	81 (67–91)	0.08	29	18	62 (46–75)	0.36
	Yes	55	26	68 (57–78)		46	35	57 (45–68)	
Alcohol##	Normal use	78	25	76 (66–84)	0.14	64	39	62 (52–72)	0.12
	Abuse	15	10	60 (39–79)		11	14	44 (24–65)	
BMI	<18,5	3	1	75 (19–99)	0.97	3	1	75 (19–99)	0.95
	18,5 to 25	52	18	74 (62–84)		41	29	59 (46–70)	
	25 to 30	27	12	69 (52–83)		22	17	56 (40–72)	
	>30	11	4	73 (45–92)		9	6	60 (32–84)	
Tumor site	Left	23	7	77 (58–90)	0.67	17	13	57 (37–75)	0.70
	Right	37	17	69 (54–80)		34	20	63 (49–76)	
	Rectum	33	11	75 (60–87)		24	20	55 (39–70)	
MSI¤	Stable	35	15	70 (55–82)	1.00	28	22	56 (41–70)	1.00
	Unstable	8	3	73 (39–94)		6	5	55 (23–83)	
Plasma volume	< 3.5 ml	52	18	74 (62–84)	0.69	43	27	61 (49–73)	0.59
	3.5 ml	41	17	71 (57–82)		32	26	55 (42–68)	

*p-value, two-sided Fisher’s exact test, *p*<0.05 considered statistically significant

*NR* Not relevant, *P* Positive, *N* Negative, *%* Positive fraction detected

#Former smokers and smokers pooled

##Abuse: Women > 7 units per week, Men >14 units per week

¤MSI determined by Immunohistochemistry, data not available on all cases

**Table 4:** Sept9 positivity of individuals with NED

		1/3 algorithm				2/3 algorithm			
Variable		*P*	*N*	% (95 % CI)	p*	*P*	*N*	% (95 % CI)	p*
		27	123	18	NR	27	123	18	NR
Sex	Female	11	64	15 (8–25)	0.4	2	73	3 (0–9)	0.44
	Male	16	59	21 (13–32)		5	70	7 (2–15)	
Age	≤65	12	86	12 (6–20)	0.015	2	96	2 (0–7)	0.05
	>65	15	37	29 (17–43)		5	47	10 (3–21)	
Hypertension	No	18	74	20 (12–29)	0.66	4	88	4 (1–11)	1.00
	Yes	9	49	16 (7–27)		3	55	5 (1–14)	
Diabetes	No	25	122	17 (11–24)	0.08	6	141	4 (2–9)	0.13
	Yes	2	1	67 (9–99)		1	2	33 (0–91)	
Arteriosclerosis	No	17	99	15 (9–22)	0.07	2	114	2 (0–6)	0.007
	Yes	10	24	29 (12–39)		5	29	15 (5–31)	
Respiratory disease	No	24	105	19 (12–26)	0.77	5	124	4 (1–8)	0.25
	Yes	3	18	14 (3–36)		2	19	10 (1–30)	
Arthritis	No	23	118	16 (11–23)	0.06	7	134	5 (2–10)	1.00
	Yes	4	5	44 (14–79)		0	9	0 (0–34)	
Smoke#	No	10	54	16 (8–27)	0.33	2	62	3 (0–11)	0.36
	Yes	17	69	20 (12–30)		5	81	6 (2–13)	
Alcohol##	Normal use	24	103	19 (12–27)	0.77	6	121	5 (2–10)	1.00
	Abuse	3	20	13 (3–34)		1	22	4 (0–22)	
BMI	<18,5	0	5	0 (0–52)	0.92	0	5	0 (0–52)	0.61
	18,5 to 25	15	67	18 (11–28)		5	77	6 (2–14)	
	25 to 30	9	39	19 (9–33)		1	47	2 (0–11)	
	>30	3	12	20 (4–48)		1	14	7 (0–32)	
Plasma volume	< 3.5 ml	20	64	24 (15–34)	0.05	6	78	7 (3–15)	0.14
	3.5 ml	7	59	11 (4–21)		1	65	2 (0–8)	

*p-value, two-sided Fisher’s exact test, *p*<0.05 considered statistically significant

*NR* Not relevant, *P* Positive, *N* Negative, *%* Positive fraction, *NED* No Evidence of Disease

#Former smokers and current smokers pooled

##Abuse: Women > 7 units per week, Men >14 units per week

Of the other variables only Arthritis and Arteriosclerosis consistently affected Sept9 outcome. Arthritis was associated with an increased false negative rate (Table 3), which was significant for the 1/3 algorithm (*p* = 0.005) and borderline for the 2/3 algorithm (*p* = 0.07). Arteriosclerosis was associated with an increased false positive rate (Table 4). This association was significant for the 2/3 algorithm (*p* = 0.007) and borderline for the 1/3 algorithm (*p* = 0.07).

Female gender was associated with an increased false negative rate for the more sensitive 1/3 algorithm but not the more specific 2/3 algorithm.

As a low sample input means less cfDNA available for analysis, it was tested whether a low plasma volume had any effect on Sept9 performance. No effect on sensitivity was observed (Fishers exact test *p* = 0.69 vs *p* = 0.59 for the two algorithms). On the opposite, a low plasma volume surprisingly seemed to produce more false positive controls (Table 4). However, this significance was probably driven by age, as more subjects aged >65 had lower plasma volumes (Additional file 3: Table S3).

### Sept9 as predictor of CRC

Logistic regression models were built to evaluate: i) how well a positive Sept9 test predicts CRC, ii) which, if any, of the available exposure variables might modify Sept9’s ability to predict CRC, and iii) the strength of the association between the Sept9 test and the diagnosis of CRC, when taking these variables into account.

First, it was evaluated which of the available variables (including symptoms) were associated with CRC. In univariate models, only Sept9 (OR 8.25, 95 % CI 4.83–14.09, *p* < 0.001), rectal bleeding (OR 2.82 95 % CI 1.76–4.5, *p* < 0.001) and Diabetes (OR 5.89, 95 % CI 1.68–20.68, *p* = 0.006) showed a significant association (Additional file 4: Table S4).

To identify variables associated with Sept9 outcome, univariate logistic regression models were built with Sept9 as outcome variable and all other variables consecutively as explaining variable. Diabetes was significant with an OR of 5.2 (95 % CI 1.4–19.1), and likewise was age when adjusted for tumor stage (OR 2.06, 95 % CI 1.1–3.8), but none of the other variables (Additional file 5: Table S5). Next, we checked if any variables modified the outcome of the Sept9 test. Age >65 and Arthritis was found to be significant modifiers with an OR of 2.46 (95 % CI 1.14–5.30) and 0.03 (95 % CI 0.00–0.22), respectively. Consequently, we allowed for effect modification from these factors in the final multivariate regression model (Additional file 6: Table S6).

All variables found to be associated with Sept9 by either Fishers exact test or regression (age, Arthritis, Arteriosclerosis, and Diabetes) were included in a final multivariate regression model with CRC as outcome. Interestingly, the adjusted OR associated with a positive Sept9 test increased from 8.25 to 29.46 (95 % CI 12.58–69.02, *p* < 0.001) for the 1/3 algorithm Table 5. Similar results were obtained for the 2/3 algorithm (Additional file 7: Table S7).

**Table 5:** Predictors of Colorectal Cancer, 1/3 algorithm

Predictor	OR (95 % CI)	*p*-value*
Sept9 -crude estimate	8.25 (4.83–14.09)	0.000
Correction for other variables	Multivariate OR (95 % CI)	*p*-value*
Sept9 -adjusted OR	29.46 (12.58–69.02)	0.000
Demographic characteristics		
Age >65	2.80 ( 1.23–6.36)	0.014
Age as effect–modificator of Sept9	0.24 (0.07–0.80)	0.020
Co-morbidities		
Diabetes	2.26 (0.51–10.05)	0.283
Arterioschlerosis	0.39 (0.18–0.86)	0.019
Arthritis	4.88 (1.30–18.42)	0.019
Arthritis as effect-modificator of Sept9	0 .02 (0.000.22)	0.001

*p<0.05 considered statistically significant

## Discussion

### Clinical performance

It is well examined that the Sept9 test can be used to identify occult CRC. More than 15.000 subjects have been tested and the reported sensitivity ranges from 36.6 to 95.6 % [14]. The test has primarily been applied to cases and controls selected from separate populations i.e. cases were typically patients with symptomatic CRC found at colonoscopy, while controls were often screening subjects or symptomatic individuals found to be tumor negative after colonoscopy. This makes statistical comparison uncertain, and furthermore does not fulfil the REMARK criteria [15]. The present study, with cases and controls selected from the same cohort of symptomatic subjects, who all had their blood samples drawn and processed according to the same standard operating procedure, fulfills the requirement of the REMARK criteria. Moreover the included subjects have gender and comorbidity distributions similar to what can be expected in a screening population. This might be the reason that the overall results are more similar to that of recent screening based cohorts than of earlier case–control studies [10, 12, 13]. The present study indicated that the Sept9 assay had low sensitivity in detecting early stage tumors (adenomas and stage I carcinomas). However, that may potentially be explained by the limited plasma volume used for analysis (≤3.5 ml). It has been reported that the number of ctDNA (cell-free tumor DNA) genome equivalents per 5 milliliter blood often is less than ten for patients with stage I carcinomas [16]. Accordingly, to increase the sensitivity towards early stage tumors it will probably be necessary to increase the plasma volume. In addition to plasma volume other factors may potentially also influence adenoma sensitivity. A recent report indicated that the methylation of the Sept9 locus is a late event in the transformation of adenomas to carcinomas [17]; even if adenomas release ctDNA, it may not be methylated and hence may not be detected by the Sept9 assay. One way to mitigate this particular problem could be to use multiple markers, including markers targeting adenomas, rather than Sept9 alone. Along these lines we hypothesize that to reach optimal sensitivity and specificity of both adenomas and early stage carcinomas an increased plasma volume and a multiplex test, targeting several colorectal neoplasia specific methylation markers, is needed.

### Factors with impact on assay performance

The influence of demographic parameters on the Sept9 test has previously only been sparsely examined. In line with other reports we showed that gender and tumor localization did not affect assay sensitivity [10, 12, 13, 18, 19]. Previously, deregulation of Sept9 expression has been reported to be associated with genomic instability by at least two mechanisms associated to chromosomal instability (CIN), namely by mitotic spindle defects and/or incomplete cell division [20]. Therefore we investigated whether Sept9 methylation was better at predicting CIN than microsatellite unstable (MSI) tumors? Surprisingly, we could not identify differences between the positivity rates for MSI and microsatellite stable (MSS) cancers (Fishers exact test, *p* = 1.00, Table 3).

The only factor besides tumor stage recurrently reported to influence Sept9 performance is age. Higher age has been described to be associated with both decreasing sensitivity and specificity [12, 13]. Our findings are in support of this observation, as we also showed the assay to have reduced specificity for the oldest test subjects (age > 65). The decreasing specificity with older age might be partly explained by the known correlation between chronological age and increased genome-wide DNA methylation changes [21], but could also be due to a higher prevalence of chronic diseases in elderly compared to younger subjects. Several studies associate various chronic diseases with DNA methylation changes [22, 23]. This might lead to a higher risk of coincident and non-CRC related methylation of the Sept9 locus in elderly subjects. Though a positive Sept9 test should not be regarded as confirmative evidence for CRC, and should always be confirmed by a colonoscopy, a decreased specificity with age >65 challenges a test aimed at subjects at age 50–75 years, and lead to larger down-stream costs. To address this problem an age-differentiated use of the two Sept9 scoring algorithms could be considered: if the 1/3 algorithm is applied to subjects ≤ 65 and the 2/3 algorithm is applied to subjects >65 years the combined sensitivity and specificity is 64 % and 89 % compared to 0.73 % and 0.82 % for the 1/3 algorithm alone (Additional file 8: Table S8).

In contrast to earlier studies, several co-morbidities influenced the Sept9 test in the present study [10, 13, 24]. Particularly, subjects with Arthritis were difficult to score correctly for the Sept9 test. This has not been reported earlier. Nevertheless, in 2008 the assay was tested in a cohort of 315 control subjects without CRC, but with different comorbidities [24]. By going through the reported data we observed, consistent with the present study, that 20 % of NED subjects with Rheumatoid arthritis were false positive and similarly that patients with Lupus also had a high false positive rate (14.2 %). Since 1966 it has been known that autoimmune inflammatory diseases such as Lupus, Polyarthritis, or Rheumatoid arthritis are associated with significant elevated levels of cfDNA [25–27]. We therefore speculate that the decreased assay sensitivity observed in subjects with Arthritis could be due to increased circulating levels of arthritis-associated cfDNA, making it difficult to detect the few copies of methylated ctDNA from the CRC. Further, a recently published study of DNA methylome changes in Rheumatoid arthritis indicates that DNA hypermethylation is a part of the disease etiology and that the methylation alterations continue to evolve as the disease progresses to chronic Rheumatoid arthritis. We consider that this dynamic pattern may lead to cancer-independent methylation of Sept9, and hence a higher false positivity rate among subjects with NED and arthritis [28].

For subjects with NED, Diabetes and Arteriosclerosis showed borderline significant association to false positive Sept9 results. Both diseases generate generalized inflammation in the body, and hence potentially increased levels of cfDNA and methylome changes, however additional studies are needed to establish this confidently.

### Strengths and limitations of our study

A major strength of this study is that it is based on a well-characterized nested case–control design, which minimizes the risk of the selection bias that was seen in several of the early Sept9 studies. The available lifestyle factors were self-reported by the patients, with the uncertainty this may cause, whereas BMI, follow-up and information about chronic diseases were collected from the medical records. In order to eliminate potential confounding effects from age and gender we matched cases and controls on these parameters. The male and female cases were further matched on tumor site and stage. Collectively this fulfills the requirements for statistical comparison of case and controls. One obvious limitation of the study is the size of the cohort, which counted only 299 subjects. Accordingly, near-significant differences between cases and controls (Type II error) may still reflect potentially interesting observations. Another limitation is that the Sept9 test is validated for 3.5 mL of plasma and only 139 subjects fulfilled this requirement. Though no significant difference was observed as a result of the lower plasma volume, this could influence especially the overall assay sensitivity. Finally, we allowed for a wide age range in our cohort with 45 subjects <50 years of age. Therefore the age of the cohort differs slightly from that of a screening cohort, where all subjects are >50. The wide age range may enhance the differences in assay performance due to age when subjects >65 are compared to subjects ≤65.

## Conclusions

In conclusion, the present nested case–control study indicates that the Sept9 assay has an overall sensitivity of 73 % and a specificity of 82 % (1/3 algorithm). While these numbers appear promising, the sensitivity for adenomas and stage I tumors was limited. Naturally, the utility of the assay for CRC population screening will require improved sensitivity for detection of these early stage tumors. We consider that increasing the plasma volume will be essential to achieve the needed improvement, but this must be tested in future studies. In addition, we showed that high age and comorbidities like Arthritis, Arteriosclerosis, and Diabetes affected assay performance negatively. Taken together this might partly explain why the performance of the Sept9 assay in recent screening based studies varies from the performance estimates of previous retrospective case–control studies. In addition, the findings indicate that age and comorbidities alter both the DNA methylome and the levels of circulating DNA in an individual. This implies that all future blood-based assays, targeting a few ctDNA copies in a large pool of cfDNA, especially methylation-sensitive assays, may be affected.

## Additional files

Additional file 1:
**Table S1.** Association between variables in cohort. *Two-sided Fisher’s exact test, all numbers are *p*-values. *p* < 0.05 considered statistically significant. # Former smokers and current smokers pooled vs non-smokers. ## Abuse: Women > 7 units per week, Men >14 units per week. ### Underweight < 18,5, Normal 18,5-25, Overweight 25–30, Heavy overweight >30. (DOC 35 kb) Additional file 2:
**Table S2.** Individuals with CRC stratified by tumorstage and age. *p*-value <0.001 Fishers exact test. (DOC 30 kb) Additional file 3:
**Table S3.** Positivity of subjects with NED stratified by plasma volume and age. * Two-sided Fisher’s exact test, *p* < 0.05 considered statistically significant Fraction: Positive fraction detected. NR: Not Relevant. NED: No Evidence of Disease. (DOC 35 kb) Additional file 4:
**Table S4.** Predictors of CRC in univariate regression, 1/3 algorithm. ¤ *p*-values for Sept9 2/3 algorithm similar (data not shown). * *p*-value < 0.05 is considered statistically significant. # Former smokers and current smokers pooled vs non-smokers. ## Abuse: Women > 7 units per week, Men >14 units per week. ### Underweight < 18,5, Normal 18,5–25, Overweight 25–30, Heavy overweight >30. (DOC 39 kb) Additional file 5:
**Table S5.** Factors associated with a positive Sept9 outcome. ¤ *p*-values for Sept9 2/3 algorithm similar (data not shown). * *p*-value < 0.05 is considered statistically significant. # Former smokers and current smokers pooled vs non-smokers. ## Abuse: Women > 7 units per week, Men >14 units per week. ### Underweight < 18,5, Normal 18,5–25, Overweight 25–30, Heavy overweight >30. (DOC 33 kb) Additional file 6:
**Table S6.** Effect modificators of Sept9 positivity in CRC. ¤ *p*-values for Sept9 2/3 algorithm similar (data not shown). * *p*-value < 0.05 is considered statistically significant. # Former smokers and current smokers pooled vs non-smokers. ## Abuse: Women > 7 units per week, Men >14 units per week. ### Underweight < 18,5, Normal 18,5–25, Overweight 25–30, Heavy overweight >30. (DOC 34 kb) Additional file 7:
**Table S7.** Predictors of Colorectal Cancer, 2/3 algorithm. * *p* < 0.05 considered statistically significant. (DOC 37 kb) Additional file 8:
**Table S8.** Sensitivity and specificity for Sept9 after age adjusted combinations of positivity algorithms. (DOC 31 kb)

## Abbreviations

CRC: Colorectal cancer
DNA: Deoxyribo-nucleic-acid
PCR: Polymerase chain reaction
CI: Confidence interval
OR: Odds ratio
FOBT/FIT: Fecal occult blood test/ Fecal immunochemical test
RNA: Ribo-nucleic-acid
HNPCC: Hereditary nonpolyposis colorectal cancer/Lynch syndrome
FAP: Familial adenomatous polyposis
TNM: Classification system for malignant tumors
DCCG: Danish colorectal cancer group
AMI: Acute myocardial infarction
NED: No evidence of disease
UICC: Union for international cancer control
EDTA: Ethylene-diamine-tetraacetic-acid
cfDNA: Circulating cell-free DNA
ACTB: Actin beta, protein coding gene used as internal control in this setting
Ct: Cycle threshold in PCR reactions
REMARK: REporting recommendations for tumour MARKer prognostic studies, article
ctDNA: Circulating cell-free tumor DNA
CIN: Chromosomal instability
MSI: Microsatellite unstable
MSS: Microsatellite stable
