# Supplementary material for: Performance of the colorectal cancer screening marker Sept9 is influenced by age, diabetes and arthritis: a nested case–control study
Source: BMC Cancer. 2015 Oct 29;15:819. doi: 10.1186/s12885-015-1832-6 (PMC4625973; doi:10.1186/s12885-015-1832-6)
Supplement: Additional file 1: — Table S1. Association between variables in cohort. *Two-sided Fisher’s exact test, all numbers are p-values. p < 0.05 considered statistically significant. # Former smokers and current smokers pooled vs non-smokers. ## Abuse: Women > 7 units per week, Men >14 units per week. ### Underweight < 18,5, Normal 18,5-25, Overweight 25–30, Heavy overweight >30. (DOC 35 kb) [file 12885_2015_1832_MOESM1_ESM.doc]

**Supplementary Table S1**

**Association between variables in cohort**

|  | **Diabetes** | **Smoke#** | **Alcohol abuse##** | **BMI###** | **Arterio-sclerosis** | **Arthritis** | **Hyper-tension** | **Respiratory disease** |
| --- | --- | --- | --- | --- | --- | --- | --- | --- |
| **Male gender** | 0.816 | **0.003** | **0.007** | **0.000** | **0.017** | 0.340 | 0.555 | 0.458 |
| **Age>65** | **0.027** | 0.252 | 0.462 | 0.235 | **0.000** | 0.275 | **0.000** | 0.077 |
| **Diabetes** | - | 0.633 | 0.543 | **0.038** | 0.764 | 0.319 | **0.000** | 0.705 |
| **Smoker#** | - | - | 0.067 | 0.107 | 0.233 | 0.137 | 1.000 | **0.022** |
| **Alcohol abuse##** | - | - | - | 0.858 | 0.447 | 1.000 | 0.644 | 0.812 |
| **BMI###** | - | - | - | - | 0.474 | 0.743 | **0.003** | 0.356 |
| **Arterio- sclerosis** | - | - | - | - | - | 0.347 | **0.002** | 0.155 |
| **Arthritis** | - | - | - | - | - | - | 1.000 | **0.032** |
| **Hyper-tension** | - | - | - | - | - | - | - | 0.568 |

*Two-sided Fisher's exact test, all numbers are p-values. p < 0.05 considered statistically significant

# Former smokers and current smokers pooled vs non-smokers

## Abuse: Women > 7 units per week, Men >14 units per week

### Underweight < 18,5, Normal 18,5-25, Overweight 25-30, Heavy overweight >30
