# Supplementary material for: Performance of the colorectal cancer screening marker Sept9 is influenced by age, diabetes and arthritis: a nested case–control study
Source: BMC Cancer. 2015 Oct 29;15:819. doi: 10.1186/s12885-015-1832-6 (PMC4625973; doi:10.1186/s12885-015-1832-6)
Supplement: Additional file 2: — Table S2. Individuals with CRC stratified by tumorstage and age. p-value <0.001 Fishers exact test. (DOC 30 kb) [file 12885_2015_1832_MOESM2_ESM.doc]

**Supplementary Table S2**

**Individuals with CRC stratified by tumorstage and age**

| **Tumor stage** |  | **Age ≤65** | **Age >65** |
| --- | --- | --- | --- |
| **Stage I** | n | 13 | 22 |
|  | % | 37.1 | 62.9 |
| **StageII** | n | 11 | 24 |
|  | % | 31.4 | 68.6 |
| **StageIII** | n | 30 | 0 |
|  | % | 100 | 0.0 |
| **Stage IV** | n | 20 | 8 |
|  | % | 71.4 | 28.6 |

p-value <0.001 Fishers exact test
