# Supplementary material for: Performance of the colorectal cancer screening marker Sept9 is influenced by age, diabetes and arthritis: a nested case–control study
Source: BMC Cancer. 2015 Oct 29;15:819. doi: 10.1186/s12885-015-1832-6 (PMC4625973; doi:10.1186/s12885-015-1832-6)
Supplement: Additional file 3: — Table S3. Positivity of subjects with NED stratified by plasma volume and age. * Two-sided Fisher’s exact test, p < 0.05 considered statistically significant Fraction: Positive fraction detected. NR: Not Relevant. NED: No Evidence of Disease. (DOC 35 kb) [file 12885_2015_1832_MOESM3_ESM.doc]

**Supplementary Table S3**

**Positivity of subjects with NED stratified by plasma volume and age**

| **Plasma volume and age** | **Positive** | **Negative** | **Fraction %** | **p-value*** |
| --- | --- | --- | --- | --- |
| n | 27 | 123 | 18 | NR |
| **Plasma < 3.5 ml** |  |  |  |  |
| Age ≤65 | 8 | 45 | 15 | 0.02 |
| Age >65 | 12 | 19 | 39 |  |
| **Plasma ≥3.5 ml** |  |  |  |  |
| Age ≤65 | 4 | 41 | 9 | 0.67 |
| Age >65 | 3 | 18 | 14 |  |

* Two-sided Fisher's exact test, p < 0.05 considered statistically significant Fraction: Positive fraction detected

NR: Not Relevant

NED: No Evidence of Disease
