# Supplementary material for: Performance of the colorectal cancer screening marker Sept9 is influenced by age, diabetes and arthritis: a nested case–control study
Source: BMC Cancer. 2015 Oct 29;15:819. doi: 10.1186/s12885-015-1832-6 (PMC4625973; doi:10.1186/s12885-015-1832-6)
Supplement: Additional file 4: — Table S4. Predictors of CRC in univariate regression, 1/3 algorithm. ¤ p-values for Sept9 2/3 algorithm similar (data not shown). * p-value < 0.05 is considered statistically significant. # Former smokers and current smokers pooled vs non-smokers. ## Abuse: Women > 7 units per week, Men >14 units per week. ### Underweight < 18,5, Normal 18,5–25, Overweight 25–30, Heavy overweight >30. (DOC 39 kb) [file 12885_2015_1832_MOESM4_ESM.doc]

**Supplementary Table S4**

**Predictors of CRC in univariate regression, 1/3 algorithm**

|  | **Crude OR (95% CI)** | **p-value*** |
| --- | --- | --- |
| Sept9 | 8.25 (4.83 - 14.09) | **0.000** |
| Male gender | 0.96 (0.61 - 1.51) | 0.862 |
| Age>65 | 1.42 (0.89 - 2.26) | 0.142 |
| Rectal bleeding | 2.82 (1.76 - 4.53) | **0.000** |
| Anemia | 0.35 (-0.27 - 0.97) | 0.264 |
| Weightloss | 0.46 (-0.04 - 0.97) | 0.071 |
| Changed defecation pattern | -0.40 (-0.86 - 0.06) | 0.092 |
| Abdominal pain | -0.18 (-0.64 - 0.28) | 0.442 |
| Palpable mass abdomen | 0.27 (-0.74 - 1.29) | 0.599 |
| Distention | -0.33 (-0.91 - 0.24) | 0.255 |
| Hypertension | 1.01 (0.63 - 1.61) | 0.963 |
| Diabetes | 5.89 (1.68 - 20.68) | **0.006** |
| Arteriosclerosis | 0.59 (0.33 - 1.07) | 0.082 |
| Respiratory disease | 0.5 (0.2 - 1.10) | 0.068 |
| Arthritis | 1.0 (0.40 - 2.60) | 0.988 |
| Smoke# | 1.14 (0.72 - 1.80) | 0.590 |
| Alcohol abuse## | 1.45 (0.80 - 2.63) | 0.220 |
| BMI### | 1.03 (0.98 - 1.09) | 0.233 |

¤ p-values for Sept9 2/3 algorithm similar (data not shown)

* p-value < 0.05 is considered statistically significant

# Former smokers and current smokers pooled vs non-smokers

## Abuse: Women > 7 units per week, Men >14 units per week

### Underweight < 18,5, Normal 18,5-25, Overweight 25-30, Heavy overweight >30
