# Supplementary material for: Performance of the colorectal cancer screening marker Sept9 is influenced by age, diabetes and arthritis: a nested case–control study
Source: BMC Cancer. 2015 Oct 29;15:819. doi: 10.1186/s12885-015-1832-6 (PMC4625973; doi:10.1186/s12885-015-1832-6)
Supplement: Additional file 5: — Table S5. Factors associated with a positive Sept9 outcome. ¤ p-values for Sept9 2/3 algorithm similar (data not shown). * p-value < 0.05 is considered statistically significant. # Former smokers and current smokers pooled vs non-smokers. ## Abuse: Women > 7 units per week, Men >14 units per week. ### Underweight < 18,5, Normal 18,5–25, Overweight 25–30, Heavy overweight >30. (DOC 33 kb) [file 12885_2015_1832_MOESM5_ESM.doc]

**Supplementary Table S5**

**Factors associated with a positive Sept9 outcome**

|  | | |
| --- | --- | --- |
| 1/3 algorithm, univariate regression¤ | | |
|  | **Crude OR (95% CI)** | **p-value*** |
| Male gender | 1.55 (0.96-2.50) | 0.072 |
| Age>65 | 1.38 (0.85-2.25) | 0.192 |
| Age>65 corrected for tumor stage | 2.06 (1.12- 3.76) | **0.019** |
| Hypertension | 0.81 (0.50-1.33) | 0.407 |
| Diabetes | 5.21 (1.42-19.13) | **0.013** |
| Arteriosclerosis | 0.97 (0.53-1.77) | 0.925 |
| Respiratory disease | 0.48 (0.21-1.07) | 0.073 |
| Arthritis | 0.70 (0.25-1.96) | 0.501 |
| Smoke# | 0.99 (0.61-1.61) | 0.983 |
| Alcohol abuse## | 0.75 (0.40-1.43) | 0.385 |
| BMI### | 1.04 (0.75-1.44) | 0.812 |

¤ p-values for Sept9 2/3 algorithm similar (data not shown)

* p-value < 0.05 is considered statistically significant

# Former smokers and current smokers pooled vs non-smokers

## Abuse: Women > 7 units per week, Men >14 units per week

### Underweight < 18,5, Normal 18,5-25, Overweight 25-30, Heavy overweight >30
