# Supplementary material for: Performance of the colorectal cancer screening marker Sept9 is influenced by age, diabetes and arthritis: a nested case–control study
Source: BMC Cancer. 2015 Oct 29;15:819. doi: 10.1186/s12885-015-1832-6 (PMC4625973; doi:10.1186/s12885-015-1832-6)
Supplement: Additional file 6: — Table S6. Effect modificators of Sept9 positivity in CRC. ¤ p-values for Sept9 2/3 algorithm similar (data not shown). * p-value < 0.05 is considered statistically significant. # Former smokers and current smokers pooled vs non-smokers. ## Abuse: Women > 7 units per week, Men >14 units per week. ### Underweight < 18,5, Normal 18,5–25, Overweight 25–30, Heavy overweight >30. (DOC 34 kb) [file 12885_2015_1832_MOESM6_ESM.doc]

**Supplementary Table S6**

**Effect modificators of Sept9 positivity in CRC**

| 1/3 algorithm, bivariate regression | | |
| --- | --- | --- |
|  | **Crude OR (95% CI)** | **p-value*** |
| Male gender | 1.47 (0.46-4.75) | 0.515 |
| Age>65 | 2.46 (1.14-5.30) | **0.021** |
| Hypertension | 1.09 (0.33- 3.58) | 0.884 |
| Diabetes | 0.18 (0.01-3.32) | 0.250 |
| Arteriosclerosis | 0.27 (0.07-1.04) | 0.057 |
| Respiratory disease | 0.57 (0.09-3.46) | 0.539 |
| Arthritis | 0.03 (0.00- 0.22) | **0.001** |
| Smoke# | 0.38 (0.11-1.27) | 0.116 |
| Alcohol abuse## | 0.74 (0.15- 3.64) | 0.718 |
| BMI### | 0.98 (0.86-1.12) | 0.787 |

¤ p-values for Sept9 2/3 algorithm similar (data not shown)

* p-value < 0.05 is considered statistically significant

# Former smokers and current smokers pooled vs non-smokers

## Abuse: Women > 7 units per week, Men >14 units per week

### Underweight < 18,5, Normal 18,5-25, Overweight 25-30, Heavy overweight >30
