# Supplementary material for: Performance of the colorectal cancer screening marker Sept9 is influenced by age, diabetes and arthritis: a nested case–control study
Source: BMC Cancer. 2015 Oct 29;15:819. doi: 10.1186/s12885-015-1832-6 (PMC4625973; doi:10.1186/s12885-015-1832-6)
Supplement: Additional file 7: — Table S7. Predictors of Colorectal Cancer, 2/3 algorithm. * p < 0.05 considered statistically significant. (DOC 37 kb) [file 12885_2015_1832_MOESM7_ESM.doc]

**Supplementary Table S7**

**Predictors of Colorectal Cancer, 2/3 algorithm**

| **Predictor** | **OR (95% CI)** | **p-value*** |
| --- | --- | --- |
| Sept9 -crude OR | 20.70 (9.08-47.19) | **0.000** |
| **Correction for other variables** | **Multivariate OR (95% CI)** | **p-value*** |
|  |  |  |
| Sept9 -adjusted OR | 117.04 (25.95- 527.91) | **0.000** |
| **Demographic characteristics** |  |  |
| Age >65 | 3.29 (1.64-6.60) | **0.001** |
| Age as effect-modificator of Sept9 | 0.07 (0.01-0.43) | **0.004** |
| **Co-morbidities** |  |  |
| Diabetes | 1.56 (0.31-7.94) | 0.594 |
| Arterioschlerosis | 0.37 (0.16-0.84) | **0.017** |
| Arthritis | 1.88 (0.60-5.92) | 0.279 |
| Arthritis as effect-modificator of Sept9 | - | - |
| * p<0.05 considered statistically significant | | |
