# Supplementary material for: Performance of the colorectal cancer screening marker Sept9 is influenced by age, diabetes and arthritis: a nested case–control study
Source: BMC Cancer. 2015 Oct 29;15:819. doi: 10.1186/s12885-015-1832-6 (PMC4625973; doi:10.1186/s12885-015-1832-6)
Supplement: Additional file 8: — Table S8. Sensitivity and specificity for Sept9 after age adjusted combinations of positivity algorithms. (DOC 31 kb) [file 12885_2015_1832_MOESM8_ESM.doc]

**Supplementary Table S8**

**Sensitivity and Specificity for Sept9 after age adjusted combinations of positivity algorithms**

|  | **Unadjusted** | | **Adjusted** | | |  |
| --- | --- | --- | --- | --- | --- | --- |
| Age cut-off |  |  | **65** | **70** | **75** |  |
| Algorithm | 1/3 | 2/3 | Age ≤ 65, 1/3  Age > 65, 2/3 | Age ≤ 70, 1/3 Age > 70, 2/3 | Age ≤ 75, 1/3 Age > 75, 2/3 |  |
| Sensitivity | 0,73 | 0,59 | 0,64 | 0,70 | 0,72 |  |
| Specificity | 0,82 | 0,96 | 0,89 | 0,87 | 0,85 |  |
| * Cutoff in shift from 1/3 to 2/3 algorithm, at the age given in bold letters, alters the overall sensitivity and specificity | | | | | |  |
